# Two years into the Brazilian Reproducibility Initiative: reflections on conducting a large-scale replication of Brazilian biomedical science

**DOI:** 10.1590/0074-02760200328

**Published:** 2020-10-23

**Authors:** Kleber Neves, Clarissa FD Carneiro, Ana Paula Wasilewska-Sampaio, Mariana Abreu, Bruna Valério-Gomes, Pedro B Tan, Olavo B Amaral

**Affiliations:** 1 Universidade Federal do Rio de Janeiro, Instituto de Bioquímica Médica Leopoldo de Meis, Rio de Janeiro, RJ, Brasil; 2 Universidade Federal do Rio de Janeiro, Instituto de Biofísica Carlos Chagas Filho, Rio de Janeiro, RJ, Brasil

**Keywords:** reproducibility, multicentre studies, replication, evaluation

## Abstract

Scientists have increasingly recognised that low methodological and analytical rigour combined with publish-or-perish incentives can make the published scientific literature unreliable. As a response to this, large-scale systematic replications of the literature have emerged as a way to assess the problem empirically. The Brazilian Reproducibility Initiative is one such effort, aimed at estimating the reproducibility of Brazilian biomedical research. Its goal is to perform multicentre replications of a quasi-random sample of at least 60 experiments from Brazilian articles published over a 20-year period, using a set of common laboratory methods. In this article, we describe the challenges of managing a multicentre project with collaborating teams across the country, as well as its successes and failures over the first two years. We end with a brief discussion of the Initiative’s current status and its possible future contributions after the project is concluded in 2021.

Over the last decade, it has become clear that common practices in experimental design and analysis, coupled with a publication system that rewards productivity over rigour, might be leading to a scientific literature that lacks trustworthiness.^1^ Both theoretical arguments^2^ ^,^ ^3^ and empirical findings of low rates of replication in preclinical^4^ and clinical research^5^ ^-^ ^7^ have drawn an unsettling picture regarding the reproducibility of published scientific findings.

This has also led to calls for reform and a strong push for transparency that has been deemed a “credibility revolution” in fields such as experimental psychology.^8^ In recent years, myriad new experimental and statistical practices have been proposed, as well as changes in cultural norms and forms of assessing scientists and their work, in a collective effort to correct the course of science towards higher credibility.^9^

Among the developments that have emerged from this reform movement are large multicentre assessments of replication. The largest one to date was the Reproducibility Project: Psychology,^10^ which was followed by similar efforts in other fields (Table I). The aim of these projects has been to perform rigorously designed replications of published studies and experiments, motivated by a need to determine the size of the reproducibility crisis - and whether it deserves to be called a “crisis” at all.^11^ The rate of successful replication of experiments in different fields can give us an idea of the credibility of findings in each of them, and possibly point to some directions for action.

It was in this context that the Brazilian Reproducibility Initiative was born. Following the precedent established by other large-scale replications, we took up the challenge of assessing the reproducibility of biomedical research in Brazil. Unlike previous efforts, we chose to focus not on a subfield or on highly cited articles, but on a representative sample of our country’s published research. Brazilian science has grown quickly in volume in recent decades,^12^ under pressure to adhere to quantitative standards of central public funding agencies such as Coordination of Superior Level Staff Improvement (Capes).^13^ This has created a breeding ground for factors thought to underlie lack of reproducibility, such as a publish-or-perish mentality that incentivises low methodological rigour.^3^ Data on reproducibility of the country’s science can inform ongoing reflections on how to assess and fund research, besides raising discussion of the issue within the scientific community.

**TABLE I:** Overview of systematic replication initiatives

Reference	Area	Recruitment	# of studies	# of authors	Study sample	Experiment selection	Replications/experiment	Replication criteria	Replication rate (%)
Errington et al.^31^	Cancer biology	Science exchange	18 (16 completed)	84	High-profile studies (2010-2012)	Main results	1	p < 0.05, meta-analysis (experiments), subjective assessment (article)	31-68
Klein et al.^32^	Psychology	Open call	12	51	*Ad-hoc* selection	*Ad-hoc* selection	36	p < 0.05 (aggregate), effect size comparison	76-85
Nosek et al.^10^	Psychology	Open call	100	270	Three psychology journals (2008)	Last study	1	p < 0.05, effect size comparison, 95%CI, meta-analysis, subjective assessment	36-47
Klein et al.^33^	Psychology	Open call	28	177	*Ad-hoc* selection (community)	*Ad-hoc* selection	57-58	p < 0.05 or < 0.0001 (aggregate)	50-54
Camerer et al.^34^	Economics	Unspecified	18	18	Two economics journals (2011-2014)	Main result	1	p < 0.05, 95%CI, meta-analysis, 95%PI	61-78
Ebersole et al.^35^	Psychology	Open call	10	64	*Ad-hoc* selection (community)	*Ad-hoc* selection	20	p < 0.05 (aggregate)	30-60
Cova et al.^36^	Experimental philosophy	Open call	40	42	Most cited + random studies (2003-2015)	First study	1	p < 0.05, subjective assessment, 95%CI	70-78
Camerer et al.^25^	Social Sciences	Open call	21	24	Nature/Science (2010 and 2015)	First study	1	p < 0.05, meta-analysis, 95%PI, “small telescopes”, default Bayes factor, bayesian mixture model	57-67

Features in the table include the form of recruitment, study and experiment selection, number of independent replications (in different labs) per experiment, criteria to define a successful replication and replication rate. Typical replication criteria include (i) statistical significance in the same direction as the original effect (p < 0.05), either for single replications or aggregates of multiple replications per experiment; (ii) the significance of a meta-analysis including the original study and the replication (meta-analysis); and (iii) the effect size being within the 95% confidence interval (95%CI) or 95% prediction interval (95%PI) of the original effect. Other approaches include bayesian models and the “small telescopes” approach (Simonsohn^37^), which analyses whether the effect is significantly smaller than what the original study would have had 33% power to detect. Replication rates are shown as a range using all the criteria that are fully based on the replication (i.e., excluding meta-analysis with the original study). For Errington et al.^31^, the lower bound counts as successful replications only those described in their editorial summary as reproducing important parts of the original paper, while the upper bound also includes those that reproduced at least parts of the original paper. For Ebersole et al.^35^, the lower bound includes those that reproduced all tested effects (including interactions), while the upper bound includes those that replicated main effects but not interactions.

Coordinating a large, collaborative, multicentre study in a country of continental dimensions, however, is not without its difficulties. In the next section, we will briefly describe the overall plan for the Initiative, before moving to a discussion of some of the challenges we have met in the first two years of the project.

The Brazilian Reproducibility Initiative

The Brazilian Reproducibility Initiative began recruiting collaborators to perform replication experiments in 2018, and currently aims to replicate between 60 and 100 experiments from Brazilian biomedical sciences. Each experiment will be performed in three laboratories from a network of 63 labs across the country. The coordinating team is based at the Federal University of Rio de Janeiro and deals with the selection of experiments, logistics, funding and data management/analysis for the project. Experiments are set to start in the second half of 2020, and are expected to finish by the end of 2021. 

The first step of the Initiative was a review of commonly used models and methods in Brazilian science, in order to identify those that could be replicated by multiple labs around the country (see https://osf.io/f2a6y/ and https://osf.io/qhyae/ for details). After selecting 10 of the most prevalent methods, we performed systematic searches of a 20-year period (1998-2017) in the biomedical literature produced in Brazil, in order to select a random sample of experiments using them (see https://osf.io/u5zdq/). At the same time, we put out an open call for Brazilian laboratories working with these methods to join the project and perform replication experiments. Based on responses to the call, we selected the three methods in the first wave of replications [(3-(4,5-Dimethylthiazol-2-yl)-2,5-Diphenyltetrazolium Bromide) (MTT) assay, reverse transcription-polymerase chain reaction (RT-PCR) and the elevated plus maze] so as to maximise the number of laboratories included (see https://osf.io/qxdjt/). We then filtered for experiments that were feasible given the infrastructure and expertise of our collaborating labs, as well as the costs involved, which led to our selection of 20 experiments for each method.

The second step was the development of replication protocols (https://osf.io/gsvy2/). Our goal was to have each lab perform what we call a “naturalistic replication”, i.e., a replication attempt based exclusively on the information available in the original article. Laboratories developed protocols independently of each other, in order to provide a realistic estimation of interlaboratory variability, and thus allow us to evaluate whether lack of replicability can be due to such variation. Based on this guiding principle, we asked replicating labs to adhere as strictly as possible to descriptions provided in the original articles, justifying any discrepancies - which can be inevitable, such as in the case of different equipment. For methodological details that were not provided in the original article, we left the decision about how to fill in the gaps to each individual lab, generating three non-identical replication protocols.

Each protocol was peer-reviewed internally, both by another replicating lab that was not performing the same experiment and by a member of the coordinating team. Sample size was calculated to provide 95% power to detect the original effect in each replication. Setting power at a higher level than usual was necessary to address the fact that effect sizes in published experiments are typically inflated, due to the combination of statistical significance thresholds and bias towards positive results.^14^ ^,^ ^15^

After addressing reviewer suggestions, most protocols are now complete, and once materials are acquired, experiments will be ready to start. In the case of those using animals, protocols have to undergo ethical approval before they can be considered finished. Every protocol will be pre-registered, and should be followed as closely as possible when the experiment is performed. Once all experimental results are in - which is currently set to happen by the end of 2021 -, we will be able to estimate the overall reproducibility rate of our sample, as well as to explore factors in the original article that can predict the successful replication of results. Detailed protocols and rationale for the Initiative can be found in the Open Science Framework (https://osf.io/6av7k/), as well as in previous publications.^16^

In the sections below, we discuss conceptual and logistical challenges in the project as we look back on its initial stages. We hope what we have learned with the Brazilian Reproducibility Initiative up to the moment will be of help to researchers interested in developing systematic multicentre collaborations in order to foster more robust science.

Recruitment of collaborators

The network of collaborating labs that will perform the replications was formed by open calls that were widely publicised online, as well as through on-site lectures at universities and scientific meetings. Any Brazilian laboratory could apply, as long as they had the necessary expertise, infrastructure and personnel to perform experiments using rodent or cell models and one or more of a set of 10 commonly used methods in Brazilian biomedical science: enzyme linked immunosorbent assay (ELISA), immunohistochemistry, RT-PCR, haematoxylin and eosin (H&E) H&E histology, MTT assay, elevated plus maze, western blot, open field exploration, flow cytometry or thiobarbituric acid reactive substances (TBARS) assay. Our first general call received 71 applications over the course of three months. We subsequently opened two additional calls to fill specific gaps in expertise, with shorter application deadlines, which recruited another 11 labs. Features of the labs that registered for the project are shown in Figure.

**Figure:**
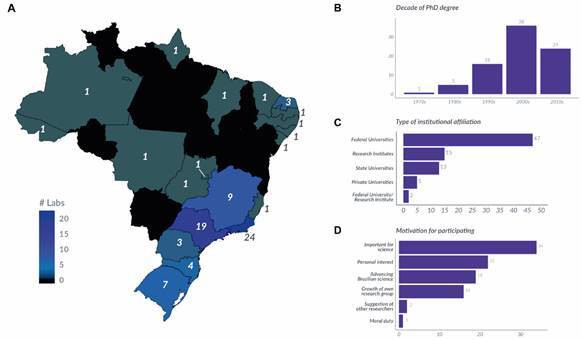
Network of registered laboratories. In our three open calls, we recruited a total of 82 laboratories, which are represented in this figure. (A) Geographical distribution of labs. (B) Career stage distribution of the researchers responsible for the labs, represented by decade of PhD degree. (C) Type of institution where labs are based. (D) Motivations for volunteering, asked as an optional open-ended question. Answers were classified in one or more common topics listed in the y-axis.

After the first set of applications was received, we selected the three methods to include in the first wave of experiments, aiming to maximise the number of participating labs; nevertheless, some labs were excluded at this stage for not working with these techniques. We provided the remaining labs with details on the expected workload, financing, authorship criteria and planned output of the project. After some withdrawals for various reasons, at the moment of writing we are set to begin experiments with a total of 63 laboratories, with 29 working with the MTT assay, 25 with RT-PCR and 17 with the elevated plus maze (some laboratories participate in more than one method). Each replicating team has between one and five members, depending on their workload. 

This means that the Initiative is currently a consortium of over 150 researchers spread across the country (A in Figure). Our initial call recruited collaborators in 19 Brazilian states plus the Federal District. Most of the labs are located in the southeast, with the states of Rio de Janeiro and São Paulo making up more than half of the participant labs, reflecting the concentration of Brazilian science within this region. Most teams are led by young researchers who obtained their PhD after 2000 (B in Figure), only half of whom are holders of National Council of Technological and Scientific Development (CNPq) research productivity fellowships - a national award for prolific researchers based on their publication, innovation and training record over the preceding five-10 years^17^ (additional data available at https://osf.io/pzbmj/). Nearly all labs are based in federal universities or research institutes (C in Figure), with only a few coming from private institutions. These features also seem to mirror the Brazilian research landscape as a whole.^18^

We expected from the start that getting scientists interested in the project would be the most important obstacle to overcome in the project. It was a positive surprise, therefore, that the number of registered labs exceeded our most optimistic predictions. When asked about their motivation, most collaborators focused on the importance of the project for science in general, for Brazilian science in particular (D in Figure) and on their personal interest in the topic. 

We hypothesise that our success in the recruitment stage was likely due to a large amount of effort put into promoting the project in scientific meetings and lectures around the country, as well as on social media. These efforts were potentiated by those of our funder, Serrapilheira Institute, a recently founded private institution that invests heavily in public outreach and is associated with renovation and outside-the-box thinking within the scientific community. Their support was vital in promoting the project through online platforms, press releases and a campaign video. In a survey of our collaborators, most of them mentioned having heard of the project by e-mail, lectures or personal contact (see data at https://osf.io/pzbmj/).

Although we cannot be certain, we also believe that the budget cuts in Brazilian science^19^ over the past few years might have influenced our recruitment process in more than one way. On one hand, they may have helped in getting a large number of teams interested, as entering a funded collaborative project allows underfunded labs to keep on working in a time of scarce resources for their own research. On the other hand, shortages of funding for students, postdocs, animal housing facilities and equipment maintenance^20^ were mentioned as reasons for withdrawing by teams who later left the project.

An additional challenge we faced was the risk that the project could be seen as potentially harmful, either for the reputation of the authors of non-replicated findings or for the public perception of Brazilian science. We were thus especially careful to promote it not as an attempt to evaluate individual results, but as a first-person effort by Brazilian science to examine itself. We also made some explicit choices to emphasise that the project will evaluate experiments and not their authors. These included not selecting more than one experiment from the same research group, blinding collaborating labs to the authors and results of the original articles, and postponing public release of the findings under replication until the end of the project. However, it is still possible that these concerns might have prevented potential collaborators from applying. These challenges are expected to return when presenting our results, when we will have to be clear that irreproducibility of research findings does not imply error or misconduct by the original authors.

Selection of experiments

Our first challenge in selecting experiments was defining what constitutes a Brazilian article. We included publications where most authors had Brazilian affiliations, including the corresponding one. This might have biased our sample towards articles with less international collaboration, but helped to select findings that were likely to have been obtained in a Brazilian laboratory. In order to have as representative a sample as possible, we performed full-text searches for our methods of interest in a random sample of life sciences articles from the Web of Science^16^ (see also https://osf.io/57f8s/). Nevertheless, included experiments were later filtered for those that could be performed with the expertise and infrastructure of our collaborating labs, as well as within our budget. This biased our sample towards cheaper and simpler experiments. As an example, around half of initially selected RT-PCR experiments used animals or primary cultures; however, due to the expertise required to dissect different tissues, only two of these remained in the final sample of 20 experiments, which consists mostly of protocols using cell lines.

A second issue in selection was our choice to reproduce a single experiment that was central to the hypothesis under study from each article (i.e., not a control experiment or a replication of previous findings). As articles came from very different fields, in many cases we did not have the expertise to make subjective judgments on the importance of experiments. We thus opted for an objective, content-independent criterion, considering experiments as central if they were explicitly mentioned in the title or abstract. Even this criterion, however, had a degree of subjectivity, leading to frequent disagreements about whether particular experiments or comparisons could be understood as a core part of the work.

We also faced the potential obstacle of whether replication experiments using animals would be considered ethically acceptable. In our understanding, performing replications on a regular basis can help avoid waste in the form of non-informative and flawed studies, which harm animals and put human subjects at risk. Nevertheless, we opted to exclude experiments that induced chronic pain or intense stress in animals for ethical reasons. The Brazilian National Council for the Control of Animal Experimentation (CONCEA) was receptive to the proposal and signalled their support for the Initiative. Still, under Brazilian law, the local committees for the ethical use of animals from the institutions where experiments will be performed are responsible for assessing and approving each replication. In this process, which is still ongoing, we have been faced with the large diversity in requirements and rules of individual committees, which we been navigating with the help of the collaborating labs.

Protocol development

As discussed, our naturalistic approach to reproducibility required us to provide each replicating lab with gaps to be filled when methodological details were not available in the original articles. This led to a need for comprehensive specification of protocol details that many researchers were not used to - many steps in laboratory procedures are never explicitly recorded, and are performed according to personal experience. We also imposed a level of pre-planning that is not common in basic research, where protocols are typically defined and adjusted as they are executed. This included explicit criteria for considering experiments as methodologically valid, in order to prevent data exclusion based on subjective judgments after experiments are done.^21^

We also chose not to contact the authors of the original publications for details, although we let them know their articles were selected for replication and intend to ask them for protocol information at a later point. According to the experiences of previous studies, contact with authors for information takes time and is not always successful.^22^ Moreover, even when the original authors are willing to help, the amount of information provided is variable, as details of published articles are often lost due to our bad track record in data management.^23^ This choice inevitably left a lot of missing information in the protocols: in some cases, gaps or ambiguities were large enough to compromise understanding of the experimental design, leading to doubts over whether we could perform a direct replication. Nevertheless, we felt that excluding these cases would lead to a biased assessment of the reproducibility of Brazilian articles, and thus chose to include them, instructing replicating labs to make their best guess about what was done in the original experiment.

Handling of these cases was further complicated by our choice to blind experimenters to the original results. As replicating teams had no access to the articles beyond the methods of the experiment in question, the coordinating team and protocol reviewers had to act as intermediaries between what the original article communicated and what the replicators received. This required a lot of back and forth communication, as well as occasional access to sections of the original articles, so that replicating labs could make an informed decision to fill in the gaps. Retrospectively, we are ambivalent about the choice of blinding replicators: although it will likely help in preventing bias, it required considerable extra effort. More importantly, it also left some of the interpretative work of protocol development in the hands of the coordinating team, potentially making the three replications less independent of each other than they would have been if teams were free to read and interpret the original articles on their own.

This is connected to another issue, one of standardisation. We actively sought to avoid overstandardisation, leaving protocol decisions to each replicating lab and preventing communication between teams working on the same protocol. On the other hand, we noticed a need for standardising some procedures, as many of the original experiments lacked appropriate control groups (such as a vehicle-treated group) or basic measures to prevent bias such as randomisation and blinding. Following our naturalistic approach, we initially left it to the replicating teams to choose which controls and measures to include. Nevertheless, during the review process, we explicitly suggested those that we deemed essential if they were not included (see Table II for a list of these measures). Still, if despite our recommendations, the replicating labs insisted that a measure or control was unnecessary, they had the final decision in this issue - although such cases were infrequent.

**TABLE II:** Recommended essential procedures

General
Sample size calculation (performed by the coordinating team) Blinded assessment of outcome
Animal studies
Randomisation of treatment assignment
Periodical assessment of animal welfare
Evaluation of anaesthesia level during surgery
Postoperative analgesia
Welfare endpoint description
Euthanasia confirmation procedure
RT-PCR
Confirmation of primer and probe designs by BLAST searches
Evaluation of RNA integrity
No-template controls to detect unintended amplification products
Criteria for use and/or exclusion of technical replicates
Balanced or randomised allocation of experimental groups in plate
MTT
Inclusion of vehicle control group (when this was not explicit)
Inclusion of blank wells
Inclusion of drug/vehicle + MTT only wells (i.e., no cells or tissue)
Inclusion of a positive control with validation criteria
Balanced or randomised allocation of experimental groups in plate

The table lists the procedures that were routinely recommended by the coordinating team for different types of experiments. These include control groups to ensure methodological validity, procedures to reduce bias and measures to minimise animal suffering. While the coordinating team deemed these standards as highly recommended, the final decision to include them was left to the replicating laboratory. BLAST: basic local alignment search tool; MTT: 3-(4,5-Dimethylthiazol-2-yl)-2,5-Diphenyltetrazolium Bromide; RT-PCR: reverse transcription-polymerase chain reaction.

Survey of beliefs and prediction markets

While working on the protocols, we also conducted a side project to investigate whether researchers are able to predict which experiments in the Initiative will have their results replicated successfully, as has been done in other replication initiatives.^24^ ^-^ ^26^ Participation was open to researchers with a background in experimental research, who could choose to predict the results of experiments with one of the three methods included in the Initiative’s first wave (for details, see https://osf.io/pjhgd/). 

Predictions were initially made through an online questionnaire that asked participants to estimate the probability of successful replication (defined as a statistically significant effect in the same direction as the original one in a meta-analysis of the three replications) and the predicted effect size for each of the 20 experiments with their chosen method. After completing the survey, participants could also join a prediction market, where they received credit to bet in the replication or non-replication of experiments, leading to live price fluctuations similar to those observed in a stock market. Previous data^24^ ^,^ ^25^ suggest that this method might lead to better performance than individual surveys, by allowing researchers to calibrate predictions according to market prices.

We recruited participants for the prediction project through an open call advertised via social media, institutional e-mails and personal communications. While we received many registrations, the completion rate for the survey was low, possibly because of the length of the process (which took around 1-2 hours to complete). This led us to open a second round of registration, in which a new round of participants completed the survey and joined the ongoing markets at the current prices. Of the 171 participants that entered the project in both stages, 71 completed the survey, and 57 traded on the markets. Participation was not as high as expected among members of the Initiative, with only 17 collaborators completing the survey, demonstrating the gap between the interest of researchers and their availability to fulfill additional deadlines. This does not reduce our belief in the potential of crowdsourced projects; however, it suggests that they require flexible designs and timelines to accommodate participants. After data collection is completed for the experiments in the Initiative, the results of the survey and markets will be analysed as one of the possible predictors of successful replication. 

Management challenges

The Initiative is a long project, planned to take place over four years (2018-2021). Getting it to finish on time is one of our biggest challenges, and maintaining a unified schedule for each of the 60+ teams has not been trivial. While every lab started together on protocol development, the work soon became unsynchronised - most labs now have finished their protocols and are ready to begin experiments, while a few still have gaps to be filled. We learned - sometimes the hard way - that we could not expect all teams to be in a similar schedule. Over the course of protocol development, we experimented with many strategies of enforcing deadlines and speculated on how to best deal with overloaded academics. Nevertheless, our tentative deadlines are still frequently adjusted once we realise they are too optimistic.

As the coordinating team, we have also tried to gather data on what the experience has been like for collaborators. An anonymous feedback survey sent to collaborators suggests that, even though most of them do see themselves as authors in the project, their impressions vary a lot, especially when it comes to deadlines. Some report the sensation of a constant workload, while others complained of long intervals between tasks due to our own delays in responding to them. Once more, this suggests that flexibility is a key element of running large-scale collaborations: individual labs have very different ways of organising their activities, and are subject to various setbacks that cannot be easily anticipated. 

We have also been making an effort to maintain regular contact with our collaborators, even during months of low workload. Most of the communication is through e-mails exchanged with each replicator team, as well as occasional online “office hours”. We also hold general meetings with all collaborators every few months, to discuss each new step of the project and to get feedback and opinions. As part of a larger effort at outreach that goes beyond the replications themselves, we also promote regular webinars with invited guests debating relevant topics in reproducibility, experimental design and methodological rigour, which are streamed via social media and available on our YouTube channel (https://www.youtube.com/channel/UCzBHLe21JbwYG7a8CcjVffA).

Ensuring that experiments can start and finish within the project’s budget is also an everyday concern. As we knew that experiments could turn out to be more expensive than initially thought, and that methodological issues might lead some of them to be repeated, we included a 40% margin of error in the initial budget. This proved to be a wise decision, as many of our costs have been driven upwards, mostly due to laboratories not possessing materials and reagents we assumed were in common use. Some cell lines were also either not available in Brazil or out of use for many years, requiring some effort to contact suppliers and/or laboratories to ask for samples. Finally, the sharp decline of the Brazilian real against the US dollar during the course of the project has also raised the price of many supplies. 

We are now delving into the challenges of data management and record keeping. With the large diversity of experiments, team compositions and experience, we are once more unable to find a one-size-fits-all strategy. Although digital records are fundamental in a decentralised project, electronic laboratory notebooks are not frequently used by our collaborators, and finding the right format for data submission is one of our current priorities. We are striving to balance the amount of information required so that the experiment is properly registered but does not overburden collaborators. That said, whether our idea of adequate registration will match those of the replicating labs remains to be seen.

Finally, the beginning of 2020 brought us a completely unexpected management challenge. Due to the coronavirus disease 2019 (COVID-19) pandemic, the vast majority of our collaborating labs have closed down, precisely at the time when protocols were finished and experiments were ready to start. With this situation, it is likely that experiments will only begin when restrictions are lifted. As this process is likely to happen heterogeneously across the country, this should lead to further desynchronisation among the progress of different labs. Prolonged shutdowns might also affect lab personnel, animal facilities and infrastructure, and may require protocols to be adapted when activities resume. While we still estimate that we should be able to finish by 2021, we are aware that results could be delayed depending on how the situation develops.

The future of the Initiative

In spite of these complications, we are reasonably confident that by 2022 the Initiative should have accomplished its goal of estimating the reproducibility of Brazilian biomedical science, at least for experiments with our chosen methods. It need not stop there, however: between our collaborating labs, our audiences in lectures and webinars, our newsletter subscribers and our followers on social media, we have connected a large network of researchers who are interested in research reproducibility. As the topic should continue to grow in importance over the next few years, it is likely that this network can be useful for the Initiative to live on beyond its initially planned goal as a systematic replication project.

Up to now, the Initiative has generated at least one spin-off project: most labs performing rodent experiments will collect intestinal samples from the animals, in order to correlate variation in gut microbiome to variation in experimental results. Although funding for this project is pending, we expect to perform it within the current structure of the Initiative. As we now have a large group of collaborators with ongoing experience in multicentre projects, it would be valuable if other collaborative efforts could arise from this network. One possible model to be followed is the Psychological Science Accelerator, an internationally distributed network of laboratories whose members vote on and organise multicentre studies.^27^ If the Brazilian Reproducibility Initiative can morph into something similar, this would fill an important gap in basic biomedical science. When done with rigour and transparency, multicentre studies are much more robust than single-lab experiments.^28^

Other possible futures for our collaboration involve going beyond experiments. Some collaborators have suggested we should place greater emphasis on education, such as online courses in data analysis, experimental design and related subjects. We currently run a “periodical” (https://peeriodicals.com/peeriodicals/brazilian-reproducibility-iniciative), where we curate a list of articles relevant to the Initiative and adjacent subjects. We have also started an online journal club affiliated to the ReproducibiliTea network^29^ to discuss articles on research reproducibility (https://reproducibilitea.org/journal-clubs/#Brasil). An interesting example to follow in the education front is the UK Reproducibility Network.^30^ This peer-led consortium has designated representatives in over 40 British universities, who promote local activities like workshops and seminars and advocate for policies that foster open and reproducible practices. With collaborators in over 40 institutions in our own country, our project could serve as a scaffold to develop a similar structure in Brazil.

Final thoughts

The Brazilian Reproducibility Initiative is still midway through its planned trajectory. Getting to this point was not simple, and the start of experiments is likely to bring a whole new set of challenges. For now, we have been using the unexpected halt brought by the COVID-19 pandemic to gear up as best as we can for the next stages of the project.

While we are satisfied with the discussion that the Initiative has generated so far, we believe it has potential to achieve much more. This, however, will depend not only on its coordinating team, but also on the many collaborators and supporters who have taken on this mission with us. At the very least, we hope that, beyond a time-stamped assessment of Brazilian biomedical science, the Initiative will leave a legacy of awareness of research credibility issues, as well as an example for other collaborative projects. In the best of scenarios, it can also become the seed of a larger effort to help promote open and reproducible science across the country.
